# Assessing oral comprehension with an eye tracking based innovative device in critically ill patients and healthy volunteers: a cohort study

**DOI:** 10.1186/s13054-022-04137-3

**Published:** 2022-09-23

**Authors:** Laetitia Bodet-Contentin, Hélène Messet-Charrière, Valérie Gissot, Aurélie Renault, Grégoire Muller, Aurélie Aubrey, Pierrick Gadrez, Elsa Tavernier, Stephan Ehrmann

**Affiliations:** 1 CHRU de Tours, Médecine Intensive Réanimation, 2 boulevard Tonnellé, Tours, France; 2 INSERM, SPHERE, UMR1246, Université de Tours Et Nantes, Tours, France; 3 CIC INSERM 1415, Tours, France; 4 CHR Orléans, Médecine Intensive Réanimation, Orléans, France; 5 CIC, Tours, France; 6 CRICS-TriggerSep FCRIN Research Network, CHRU Tours, CIC INSERM 1415, Médecine Intensive Réanimation, Tours, France; 7 Centre d’étude des pathologies respiratoires, U1100, INSERM, Université de Tours, Tours, France

**Keywords:** Language comprehension test, Critical care, Intubation

## Abstract

**Purpose:**

Communication of caregivers and relatives to patients is a major difficulty in intensive care units (ICU). Patient’s comprehension capabilities are variable over time and traditional comprehension tests cannot be implemented. Our purpose was to evaluate an oral comprehension test adapted for its automatic implementation using eye-tracking technology among ICU patients.

**Methods:**

Prospective bi-centric cohort study was conducted on 60 healthy volunteers and 53 ICU patients. Subjects underwent an oral comprehension test using an eye-tracking device: Their results and characteristics were collected. The total duration of the test was 2 and a half minutes.

**Results:**

While performing the test, 48 patients (92%) received invasive ventilation. Among healthy volunteers, the median rate of right answers was very high (93% [interquartile range 87, 100]), whereas it was lower (33% [20, 67]) for patients. For both groups, a significantly lower right answers rate was observed with advancing age (67% [27, 80] vs. 27% [20, 38] among patients and 93% [93, 100] vs. 87% [73, 93] among healthy volunteers, below and above 60 years of age, respectively) and in case of lack of a bachelor’s degree (60% [38, 87] vs. 27% [20, 57] among patients and 93% [93, 100] vs. 87% [73, 93] among healthy volunteers). For patients, the higher the severity of disease was, the lower the rate of correct answers was.

**Conclusion:**

The eye-tracking-adapted comprehension test is easy and fast to use among ICU patients, and results seem coherent with various potential levels of comprehension as hypothesized in this study.

**Supplementary Information:**

The online version contains supplementary material available at 10.1186/s13054-022-04137-3.

## Introduction

Communication of caregivers and relatives to patients is a major issue in intensive care units (ICU). It is essential that the patient be able to understand and take part in his care. Caregivers are facing several difficulties when communicating with ICU patients. Indeed, the comprehension and understanding abilities of patients are heterogeneous and vary over time due to progressive recovery from initial cerebral injury prompting ICU admission, delirium, recovery from sedative drugs and side effects of drugs used in the ICU. Furthermore, the presence of a tracheal tube precluding speech and reduced patients mobility impede patient feedback [1]. Those communication issues contribute to make the ICU stay an extremely aggressive experience for patients leading to psychological and emotional distress [2–4] and long-term psychiatric sequelae [5] in the framework of the post-ICU syndrome [6, 7]. Evaluating patients’ comprehension abilities is a key element to enable proper communication. Whereas validated scales exist to assess pain [8–10], delirium [11, 12], level of sedation or agitation [13, 14], comprehension capabilities are not easily evaluated in the ICU, in particular because this requires to evaluate patients’ feedback. To the best of our knowledge, no specific test evaluating patients’ comprehension capabilities is available to be easily implemented in the ICU. We adapted a validated test, already used by speech therapists outside the ICU to evaluate oral comprehension capabilities [15, 16], to be used by intubated ICU patients through an eye-tracking technology-based interface.

The objective of our study was to assess the feasibility of this oral comprehension test using eye tracking in healthy volunteers and ICU patients.

## Material and methods

### Study design and population

This multicentric study was carried out in two ICUs and one clinical investigation center for healthy volunteers, in France. Inclusions took place between March 7, 2019, and September 25, 2020.

The healthy volunteers were included if they were over 18 years old and French speakers. Recruitment was distributed across age ranges to proportionally represent all age groups. Twenty volunteers aged 18 to 39 years, twenty aged 40 to 65 and twenty over 65 were included. ICU patients were included if they were over 18 years old, French speakers, calm and awake as evaluated by the Richmond Agitation and Sedation Scale (RASS) score [13] between − 1 and + 1, if they had no known neurological disorder prior to hospitalization and had proper hearing and vision with correction if needed. Patients undergoing invasive mechanical ventilation or breathing spontaneously were included. The non-inclusion criteria were limited to patient or healthy volunteer under legal protection or who refused participation. All participants gave informed consent to participate in the study. Participant blinding was not possible due to the very nature of the intervention. The study protocol was approved by the regional ethics committee (Espace de reflexion éthique région centre val de Loire, Tours, France, no. 2018_090) in accordance with national regulations. The protocol was registered (clinicaltrials.gov: NCT05078632, 2021/10/14, retrospectively registered).

### The device

**Fig. 1:**
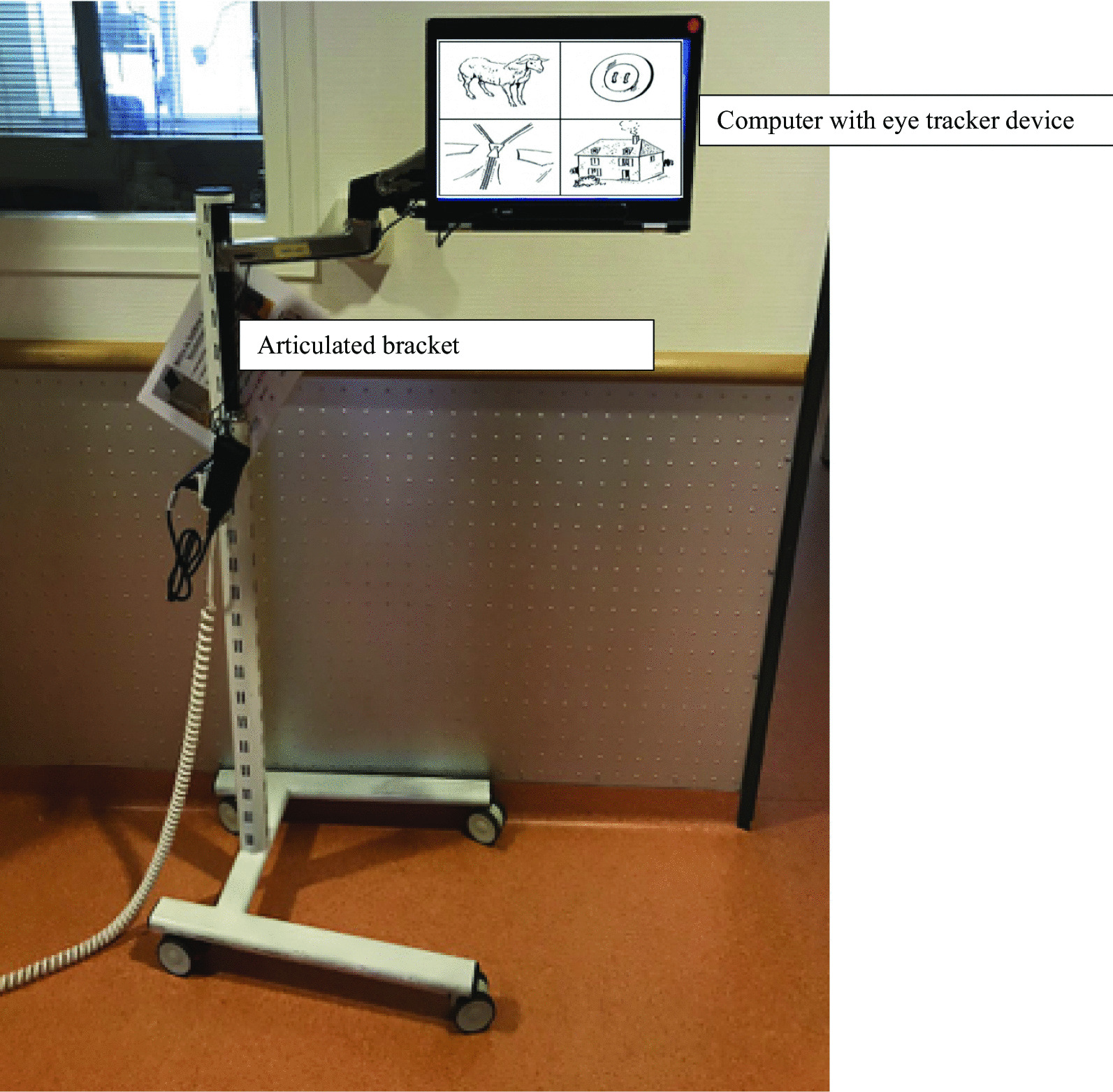
Device used to perform the test

Eye-tracking technology is used in several fields like medicine [17–21], surgery [22, 23], psychology or aerospace [24]. This technology relies on a video-based eye tracker which determines gaze direction by measuring the position of the corneal reflection of an infrared light relative to the pupil. These reflections are then analyzed to determine with a high degree of accuracy the gaze motion. This enables to calculate in real time gaze motion over the computer screen and determine what the user is looking at on the screen. We specifically developed a device: a computer with an eye-tracker device disposed on an articulated bracket in order to be ideally adjusted in front of ICU patient (Fig. 1). The screen must be placed so that it can detect the eye position and perform a visual calibration, at 60–80 cm of the patients face. Calibration (measuring characteristics of the user’s eyes to derive gaze calculation based on a physiological 3D eye model) was performed on five gaze positions on the screen [25]. The eye tracker frequency was 60 Hz, enabling the sensor to catch 60 corneal reflections per second. The eye tracker used in our study was the Tobii X2-30 compact (Tobii Pro, Danderyd, Sweden), and data were analyzed with Tobii Pro Studio (Tobii Pro).

### The comprehension test

The Montreal Toulouse test (MT-86) is a neuropsychological test created to assess language disorders [26]. It comprises 19 examinations and lasts 3 h when performed comprehensively by a speech therapist. It evaluates different components of oral comprehension. The oral comprehension test is evaluated with 47 sheets of pictures. For the present study, with the help of a team comprising speech therapists and ICU physicians and nurses, we specifically developed an adapted version of the test implemented in the eye-tracking device to be used with ICU patients. We divided the different sheets into three tests (15 sheets of pictures for each) to enable the reuse of the test at different moments during the patient’s hospital stay without learning phenomena. Each 15 sheets test evaluated three levels of comprehension: words (lexical comprehension), simple sentences (active sentence comprehension) and complex sentences (for example: long sentence, passive sentence comprehension). Each sheet contained 2 to 4 pictures as in the original test, some plates evaluating lexical comprehension comprising originally 6 pictures were simplified to 4 pictures and had been digitalized. A registered human voice was used to instruct patients in order to be as close as possible to reality considering the prosodic aspects of comprehension. Only the original pictures of the MT-86 were used without any modification.

### Test procedure

**Fig. 2:**
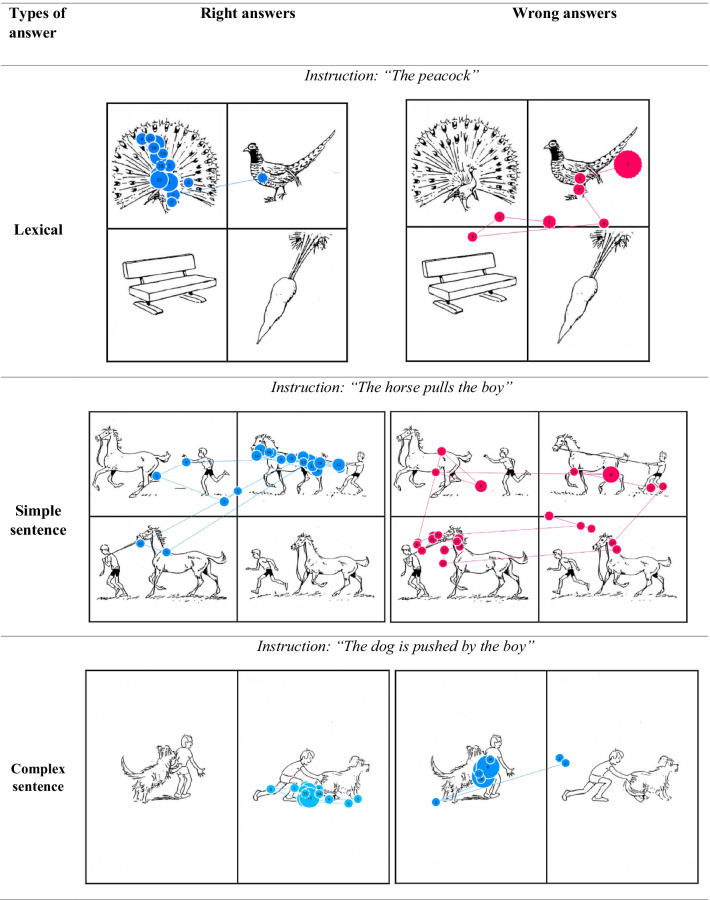
Example of instructions and answers. Each circle corresponds to a gaze fixation. The circle diameter is proportional to the fixation duration, and the numbers indicate the order of gaze fixation. Lines indicate gaze motion between two fixations

Patients underwent the test over three consecutive weekdays (one test a day, always in the same order) with a research nurse or a speech therapist (called here the assessor) according to their availability. The healthy volunteers passed the 3 tests in a room with a research nurse, with a 5-min break between each test (always in the same order). The assessor placed the computer in front of the participant, and the screen position was adjusted. After a successful calibration, the comprehension test automatically began. Several sheets with pictures were presented to the participant with vocal instruction concerning the pictures to look at (Additional file 1). The instruction given to participants was: “watch as long as possible the pictures as asked by the voice,” for example, “the peacock” or “the horse pulls the boy” (Fig. 2). During the instruction time, a white square on a black background was displayed on the screen, and thereafter, the picture sheet was presented during 6 s to the participant on the screen and gaze motion was recorded. Transition between sheets was automatic. The total duration of the test was 2 and a half minutes. During the test, the assessor was placed behind the participant to pick up any particular event which occurred impeding proper test completion. Furthermore, a camera recorded the patient’s face allowing to identify eye closure induced gaze-tracking deficiencies.

### Gaze movement analysis

Every test sheet was divided into different areas of interest, including the area of the right answer, and other areas with distractors (Additional file 2). For instructions comprising an object, several distractors were used: semantic distractors (a word with a close meaning), phonologic distractors (a word with a close sound in the French language) and distractors without any link with the answer. For instructions including a whole sentence with a situation to recognize, the distractors contained a close but different action and/or subject. For each sheet and participant, the total time spent watching the sheet and the total time of gaze fixation on the right picture were calculated in order to define the gaze time proportion spent on the right picture. The answer to each sheet was classified as “right,” “wrong” or “not interpretable.” An answer was classified as right if the subject watched the sheet for more than 3 s (half of the 6 s total sheet presentation time) and if he/she spent more than 50% of this sheet watching time, with the gaze on the right picture. If a subject spent less than 50% of the sheet watching time on the right picture, the answer was classified as wrong. We considered an answer as not interpretable if the subject spent less than 3 s watching the sheet presented. (Those cutoff values were derived from preliminary feasibility evaluations.)

### Data collection

For all participants, demographic information was gathered (age, gender, right or left-handed, education level), medical history (hearing, visual or neurological impairment), and current medication. For patients, additional information was collected: CAM-ICU scale (Confusion Assessment Method for the ICU) [11, 12], Simplified Acute Physiology Score II (SAPSII), primary diagnosis [27, 28] and organ support during ICU stay and at the time of performing the test (ventilation, dialysis, norepinephrine, etc.).

### Statistical analysis

Groups were described with absolute numbers and percentages for qualitative variables, and median and interquartile range for quantitative variables. For each participant, the rate of right answers for each test was defined as the percentage of sheets with the right answer among the sheets which were watched (with a gaze motion recorded). Boxplots were used to represent right answers among subgroups based on age, education level, SAPSII at inclusion, mode of ventilation and sedation, comprehension level evaluated by each sheet. We performed Wilcoxon tests to compare the different populations. All the analyses were performed using the software R [29]. Given the lack of gold standard to evaluate oral comprehension among ICU patients, we hypothesized a priori that patients who were older, with lower education level, higher severity of disease, ventilated or who had received sedation would potentially have lower oral comprehension capabilities, also comparatively to healthy volunteers. A higher right answer rate among those subpopulations would cast doubt about the validity of the test, whereas lower rates would appear coherent in the framework of construct validity.

## Results

**Fig. 3:**
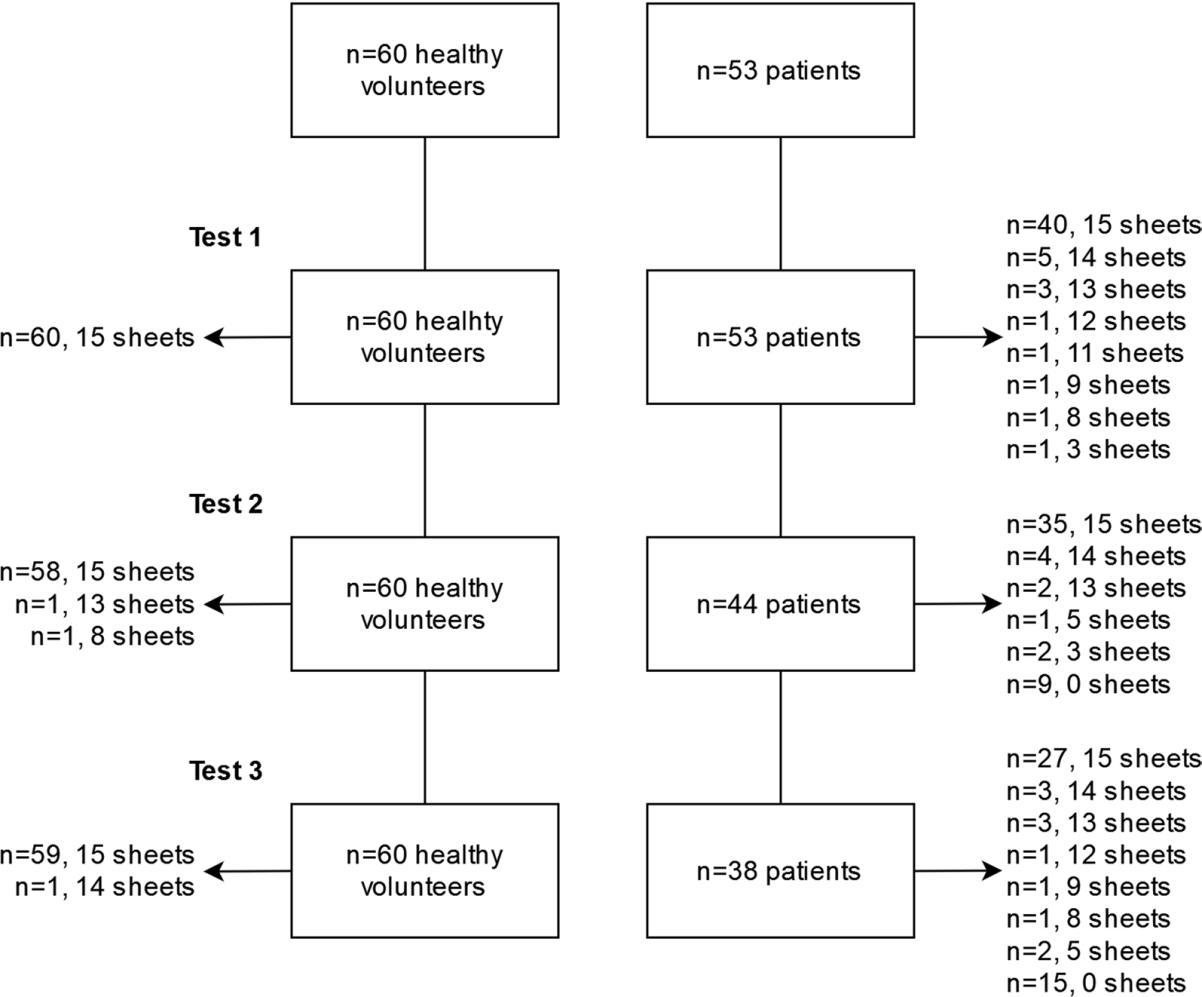
Flowchart. Numbers of sheets per test undergone by patients and healthy volunteers are presented. Tests 1, 2 and 3 were always performed in the same order. “*n* = 1, 13 sheets” means that there is one patient for who we had 13 sheets over the 15 with a gaze motion recorded

A total of 60 healthy volunteers and 53 ICU patients were included. The flowchart is presented in Fig. 3. The population characteristics are presented in Tables 1 and 2. The median age of the whole population was 59 years [41, 71]. Thirty-nine healthy volunteers (70%) and 13 patients (27%) had a bachelor’s degree. Forty-eight patients (92%) received invasive mechanical ventilation, 42 (86%) received sedation, and 19 received vasopressors (40%) during hospitalization. Patients’ population had a median duration of midazolam use of 2 days [1, 5] and a median duration of propofol use of 1 day [0, 2]). Only one patient had a positive CAM-ICU test. All healthy volunteers passed the three tests and watched the 15 sheets in each test (180 test performances). For 3 of them (2 in the test 2 and 1 in the test 3), there was a lack of gaze motion registration during the presentation of some sheets. (A total of eight sheets were concerned.) All patients passed the first test but not all underwent the other two because some of them were transferred to another ward, died before the subsequent tests or because of the unavailability of the research personnel.

**Table 1:** Characteristics of the population

	Healthy volunteers	Patients
*N*	60	53
Age (years) (Median [IQR])	54 [27, 70]	64[48, 71]
Sex Male (%)	21 (35)	33 (62)
Bachelor’s degree (%)	39 (70)	13 (27)
Laterality (%)		
Ambidextrous	2 (3)	2 (4)
Right-Handed	56 (93)	46 (89)
Left-Handed	2 (3)	4 (8)
Hearing Aid (%)	4 (7)	2 (4)
Visual Aid (%)	44 (73)	41 (77)
Eyeglasses (%)	42 (96)	41 (100)
Neurological History (%)		
None	58 (97)	40 (76)
Strokes	1 (2)	8 (15)
Neurodegenerative Pathology	0 (0)	3 (6)
Head Injury	0 (0)	1 (2)
Tumor	1 (2)	1 (2)
Psychiatric History (%)		
None	57 (95)	45 (85)
Bipolar Disorder	1 (2)	4 (8)
Depressive Syndrome	2 (3)	3 (6)
Anxiety Disorders	0 (0)	1 (2)
Previous ICU Hospitalization (%)	2 (3)	7 (13)
Usual Treatment by Benzodiazepines (%)	0 (0)	3 (6)
Usual Treatment by Neuroleptics (%)	0 (0)	0 (0)

*IQR* interquartile range

**Table 2:** Characteristics of the patients during their hospital stay

	Patients
*N*	53
Length of Stay (Median in days [IQR])	14 [8, 22]
Main Admission Diagnosis (%)	
Cardiac Arrest	1 (2)
Shock	5 (10)
Coma	9 (18)
Respiratory Failure	26 (49)
Trauma	1 (2)
Respiratory Failure from Neurological Causes	7 (13)
Other	4 (7)
SAPS II Score (Median [IQR])	37 [27, 50]
Sedation During ICU Stay (%)	42 (86)
Midazolam (%)	40 (82)
Propofol (%)	11 (22)
Invasive Ventilation (%)	48 (92)
Duration of Mechanical Ventilation (Median Days [IQR])	11 [6, 14]
Non Invasive Ventilation (%)	26 (51)
High Flow Oxygen Therapy (%)	14 (27)
Standard Oxygen (%)	39 (75)
Intermittent Renal Replacement Therapy (%)	3 (6)
Vasopressors (%)	19 (40)
Extra Corporeal Assistance (%)	1 (2)

*IQR* interquartile range

**Fig. 4:**
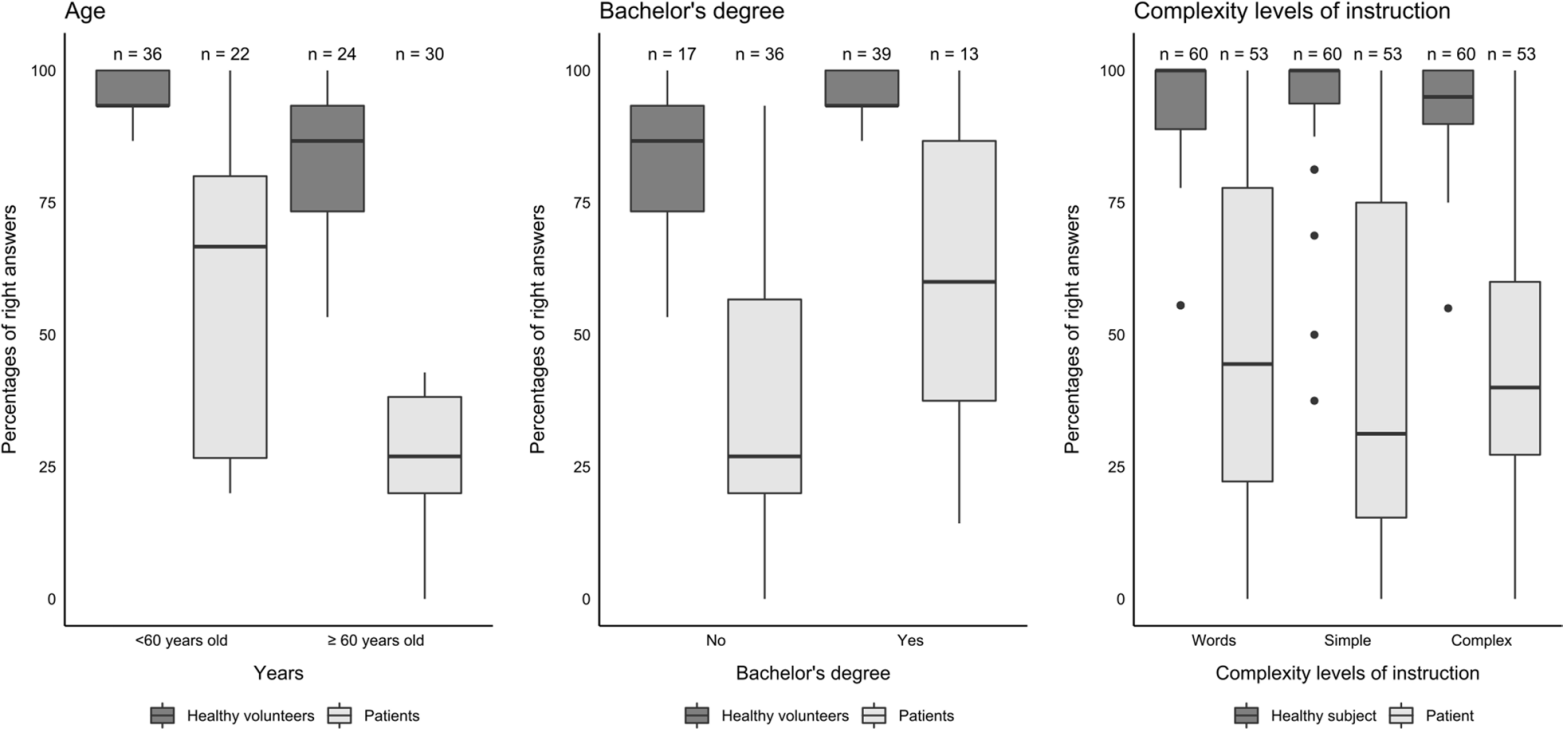
Boxplots representing percentage of right answers for the test 1 according to age, bachelor’s degree and different levels of instruction complexity

For the test 1, among healthy volunteers, the rate of right answers was excellent, with a median of 93% [87, 100] of the sheets answered correctly. It was slightly lower in the oldest age group (87% [73, 93], *p* = 0.004). The results were higher among healthy volunteers owing a bachelor’s degree (median rate of right answers 93% [93, 100] vs 87% [73, 93], *p* = 0.002). The results depending on the complexity level of the instructions (“complex sentence” *vs.* “word”) were significantly different (*p* = 0.013) in this group. The results are presented in Fig. 4. For the test 2 and the test 3, the results were similar and are presented in Additional file 3 and Additional file 4. Progressively the healthy volunteer’s test results improved over time with a median rate of right answers of 93% [87, 100], 97% [93, 100] and 100% [93, 100] for the first, second and third test (see Additional file 5).

For the test 1, among ICU patients, the global median rate of right answers was lower (38% [27, 68]) than among healthy volunteers. There was a clear decrease in right answers rate with advancing age in particular after the age of 60 (67% [27, 80] before 60 and 27% [20, 38] after 60 years of age, p = 0.004) (Fig. 4). A significant difference in the rate of right answers was observed according to education level, with patients owing a bachelor’s degree showing higher right answers rates (median rate 60% [38, 87] vs 27% [20, 60], p = 0.023) (Fig. 4). The right answer rate was not significantly different between intubated patients and patients breathing spontaneously (p = 0.23) (Additional file 6). Similarly, no significant difference in right answer rate was observed between patients who received sedation or not during the test or within 48 h before (median rate of right answers 27% [20, 70] vs. 33% [20, 67] *p* = 0.72, Additional file 6). Patients with a more severe condition at admission, as measured by a higher SAPSII, had worse results than the other: The rate of right answers of patients with a SAPSII higher than the population median of 37 was 27% [20, 40] versus 57% [27, 80] for those with a SAPSII lower than 37, *p* = 0.06. For the test 2 and the test 3, the results were similar and are presented in Additional file 3 and Additional file 4.

## Discussion

Using an oral comprehension test implemented in an eye-tracking technology-based device seems feasible among ICU patients. Indeed, the mean duration of the test is short: 2 min 33 s. The test automaticity makes it easy to use in the ICU as apart from placing the equipment in front of the patient and calibrating the device, there is no other time-consuming handling needed. Moreover, this test is usable among intubated patients including those with high severity of disease. This is especially relevant as the most severely ill patients seem to have most communication difficulties [2, 4]. Among the 60 healthy volunteers, there were only 3 defects in gaze motion registration concerning some sheets of 3 tests over the 180 test performances. These defects concerned the tests 2 and 3 only. One of the possible explanations is that the movement of the computer by the assessor between each test impaired the optimal position between the eyes of the subject and the eye tracker (after reviewing the video records, we noticed that the assessor moved sometimes the computer to start the subsequent test 2 and then 3). Considering the entire population, there were no technical issues reported. Furthermore, in patients, the results seem coherent with various levels of comprehension as compared with healthy volunteers. The older and the subjects with a lower educational level had worse results. In patients, the more severely ill had worse results which is consistent with what could be awaited. However, intubated patients and patients who had received sedatives performed equally the test, contrarily to our a priori hypothesis. However, taken together these results suggest that this kind of comprehension test implemented in the eye-tracking device could assess comprehension in this ICU population.

Our study has some limitations. First, not all the patients passed every test because of their transfer in other wards before completing the 3 tests or because of the availability of the research personnel. Second, our study included patients with a short duration of sedation which may preclude generalizability to patients with longer sedations durations. Third, there was only one patient who had a positive CAM-ICU test which is much lower than the prevalence of ICU delirium in other cohorts, and thus, results cannot be extrapolated to patients with delirium.

Fourth, there were some lack of gaze records for some sheets. For the healthy volunteers, it could probably be related to the movement of the computer by the assessor to start the subsequent test. A systematic check of the proper position between the screen and the subject for each new test seems to be important. For the patients, these defects on gaze motion records are probably related to defects in sampling frequency as the reviewing of the videos did not show any test disruption but an absence of effective gaze route drawing during the presentation of some sheets without any computer displacement. As high-performance eye trackers with a frequency as high as 2000 Hz are available, a more efficient sensor could be interesting to use. In our study, we used a 5-dot calibration to simplify the procedure, but the device allows us to use up to 9 dots. This could be an area of improvement of the gaze detection process. Fifth, we did not perform evaluation of different durations of presentation of a sheet which was always 6 s, with a fixed criteria of 3 s on the correct region of interest to validate the answer as correct. One may hypothesize that some patients may need more than 6 s to identify the correct answer. However, this could be indicative of some comprehension deficit, given that with the original paper test correct or incorrect answers are usually given very quickly in less than 2 s. Longer presentation durations would lengthen the overall test duration and thus reduce feasibility but could be evaluated in the future to clarify this point. Similarly, further evaluation of repeatability of the adapted version of the test could be useful. Finally, some voice misunderstanding issues may have occurred, potentially related to the registered male voice used. Indeed, the characteristics of the male voice are different from those of the female voice [30, 31]. Generally, a female voice is clearer than a male voice, especially in the presence of background noise, due to its frequency. Furthermore, the intelligibility of a speech depends on several aspects such as articular precision, relevance of prosodic accents or appropriate pause in the speech. These aspects are artificial in an instruction with a voice recorded. The registered voice is also an added difficulty since the loudspeaker of the tablet computer deletes or affects some of its frequencies. An optimization of the registered voice for a better intelligibility could be a way of improvement.

The results of our study offer several prospects in the field of communication in the ICU even if various aspects of the device need to be improved.

It is encouraging to notice that the automatization of the test with this device allows an easy and fast use at the bedside. A generalization of its application by a non-dedicated staff and without training could be interesting for a quick evaluation of a patient comprehension. Such standardized quick assessment could help early detection of comprehension difficulties and make it possible the implement corrective measures very early. It could also guide healthcare staff to use appropriate tools to interact with the patients. Some authors have tried to develop algorithms to guide caregivers for the choice of assistive communication tools with intubated patients according to some of their abilities (like consciousness, cognitive level, motor ability, for example) [32, 33]. The device tested in this study may enable to integrated comprehension capabilities in such an algorithm. 

## Conclusion

The oral comprehension test implemented in the eye-tracking-based device seems easy to use in the ICU and gives a result in less than 3 min. The device is reliable as there were no major technical issues during the testing. The results seem coherent with various potential levels of comprehension as compared with healthy volunteers, education level and age and thus can be considered as minimally validated.

### **Take Home message**

Implementing an oral comprehension test using an innovative eye-tracking-based interface seems feasible in critically ill intubated patients. Test results appeared coherent with various potential levels of comprehension in patients and healthy volunteers, thus validating the proof of concept of this innovative technique which requires extensive validation.

## Supplementary Information

**Additional file 1. **A video of a healthy volunteer’s test. The red dot corresponds to the gaze position

**Additional file 2. **Example of a simplified MT-86 sheet divided into different areas of interest (AOI).

**Additional file 3.** Results for test 2 according to age, bachelor’s degree and different levels of instruction complexity (a) and according to SAPSII, invasive ventilation and sedation (b).

**Additional file 4.** Results for test 3 according to age, bachelor’s degree and different levels of instruction complexity (a), and according to SAPSII, invasive ventilation and sedation (b).

**Additional file 5. **Evolution in time of the results for the healthy volunteers and patients.

**Additional file 6.** Results for the test 1 according to the SAPS II score, invasive ventilation, and sedation in patients.

## Data Availability

The datasets used in this study are available from the corresponding author on reasonable request.
