# Supplementary material for: Assessing oral comprehension with an eye tracking based innovative device in critically ill patients and healthy volunteers: a cohort study
Source: Crit Care. 2022 Sep 23;26:288. doi: 10.1186/s13054-022-04137-3 (PMC9508751; doi:10.1186/s13054-022-04137-3)

**Online Resource 2**

Title: Assessing critical oral comprehension with an eye tracking based innovative device in critically ill patients and healthy volunteers: a cohort study

**Authors**

Laetitia Bodet-Contentin, Hélène Messet-Charrière, Valérie Gissot, Aurélie Renault, Grégoire Muller, Aurélie Aubrey, Pierrick Gadrez, Elsa Tavernier, Stephan Ehrmann

**Example of a simplified MT-86 sheet divided into different areas of interest (AOI)**


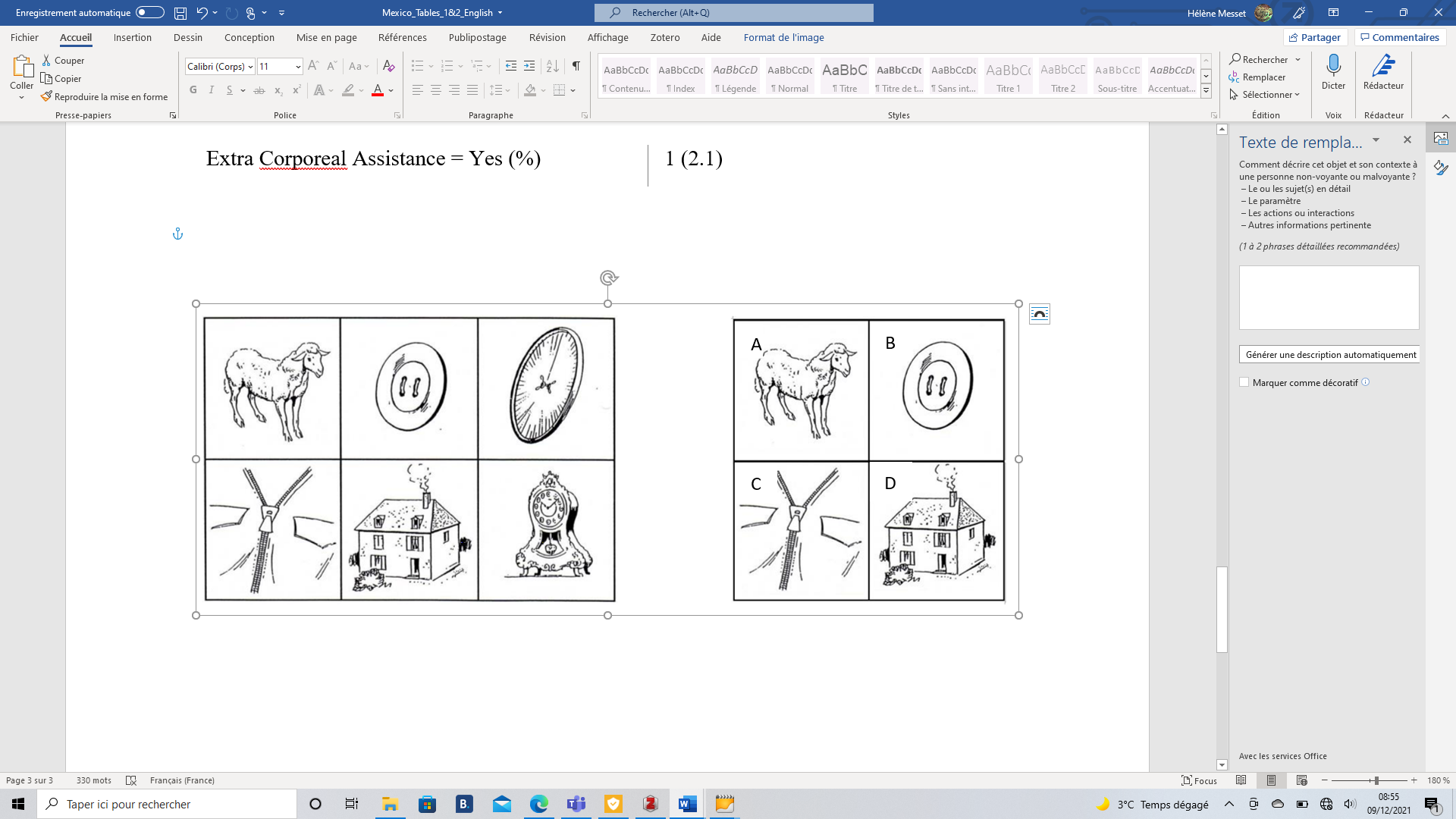


AOI A: the phonologic distractor in French language

AOI B: the right answer

AOI C: the semantic distractor

AOI D: the picture “house” has no link with the answer “button”


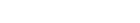

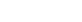

Supplement: Supplementary file 2 — Additional file 2. Example of a simplified MT-86 sheet divided into different areas of interest (AOI). [file 13054_2022_4137_MOESM2_ESM.docx]
