# Supplementary material for: Assessing oral comprehension with an eye tracking based innovative device in critically ill patients and healthy volunteers: a cohort study
Source: Crit Care. 2022 Sep 23;26:288. doi: 10.1186/s13054-022-04137-3 (PMC9508751; doi:10.1186/s13054-022-04137-3)

**Online Resource 4**

Title: Assessing critical oral comprehension with an eye tracking based innovative device in critically ill patients and healthy volunteers: a cohort study

**Authors**

Laetitia Bodet-Contentin, Hélène Messet-Charrière, Valérie Gissot, Aurélie Renault, Grégoire Muller, Aurélie Aubrey, Pierrick Gadrez, Elsa Tavernier, Stephan Ehrmann


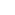

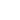

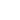


**Results for test 3 according to age, bachelor’s degree and different levels of instruction complexity (a), and according to SAPSII, invasive ventilation and sedation (b)**


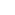

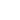

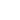

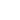


(a)


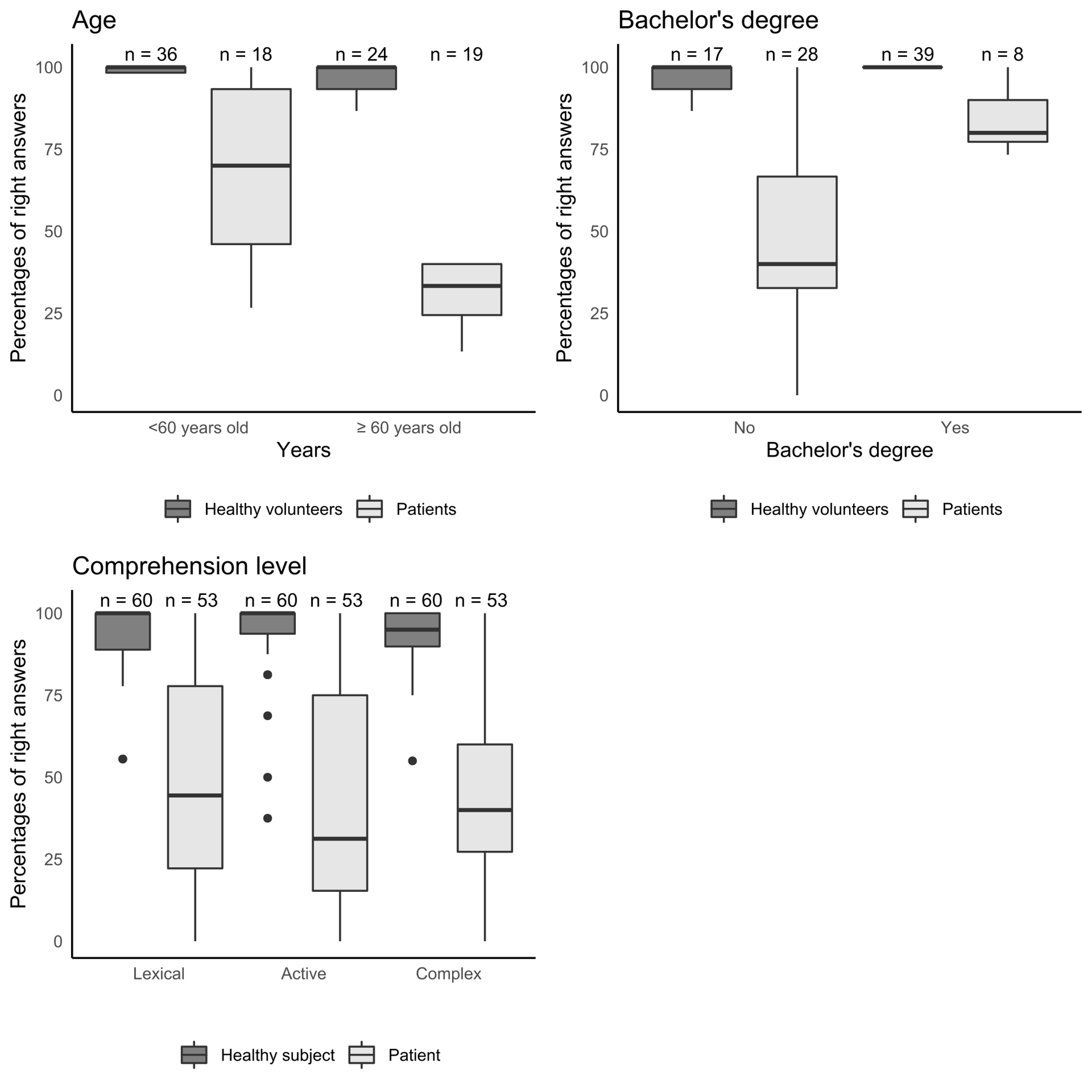

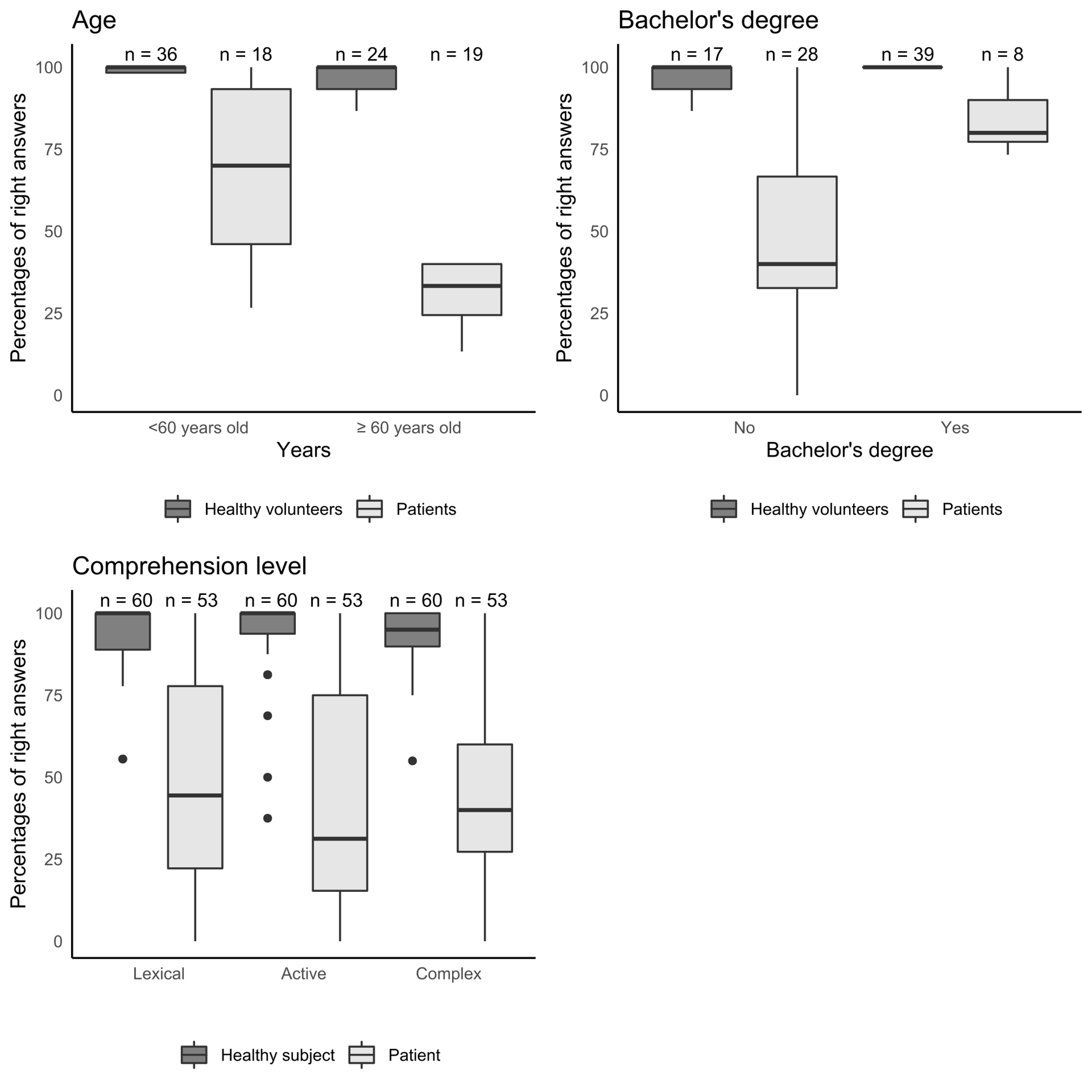


Words Simple Complex

**Complexity levels of instruction**

(b)


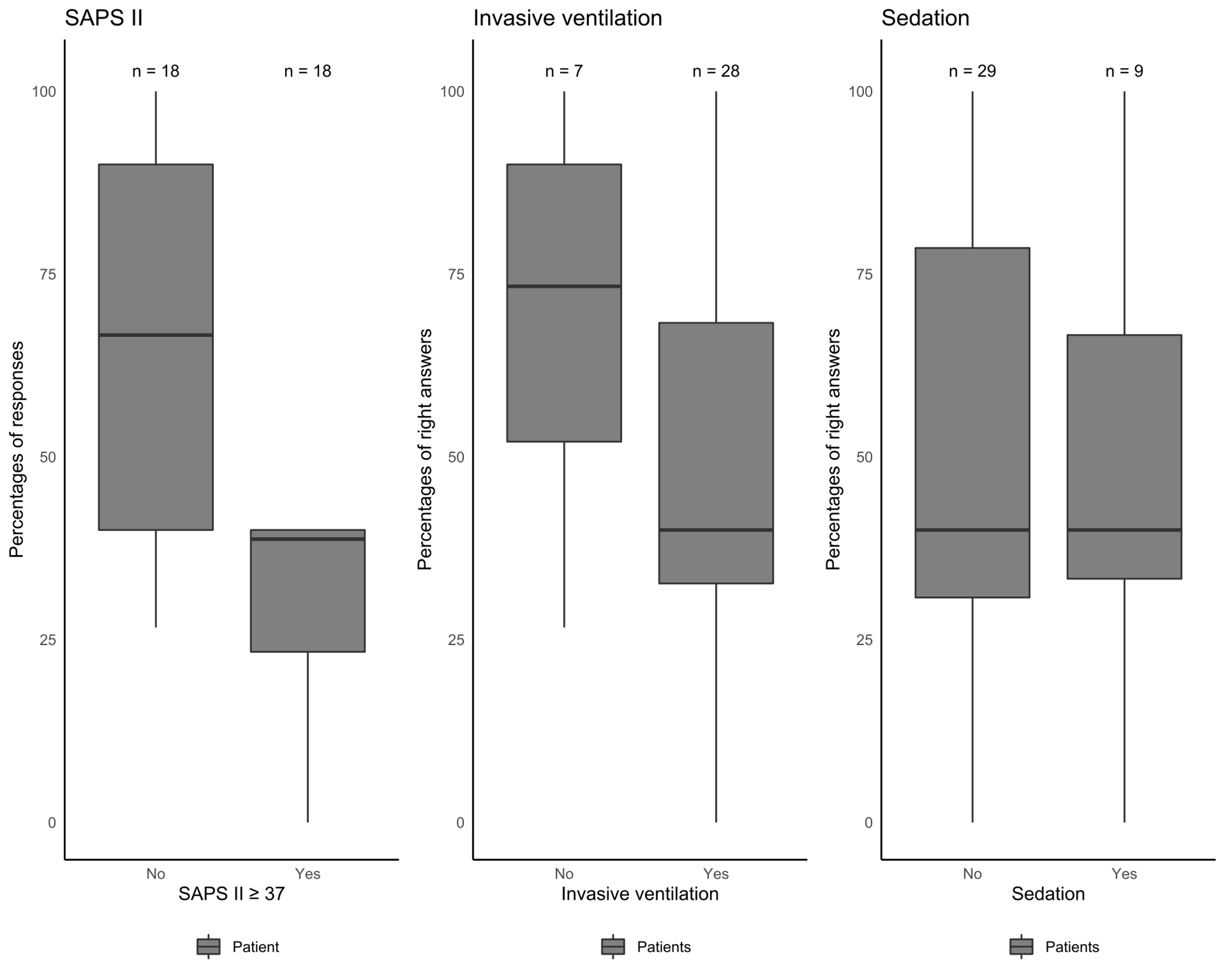


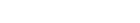

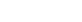

Supplement: Supplementary file 4 — Additional file 4. Results for test 3 according to age, bachelor’s degree and different levels of instruction complexity (a), and according to SAPSII, invasive ventilation and sedation (b). [file 13054_2022_4137_MOESM4_ESM.docx]
