# Supplementary material for: Assessing oral comprehension with an eye tracking based innovative device in critically ill patients and healthy volunteers: a cohort study
Source: Crit Care. 2022 Sep 23;26:288. doi: 10.1186/s13054-022-04137-3 (PMC9508751; doi:10.1186/s13054-022-04137-3)
Supplement: Supplementary file 5 — Additional file 5. Evolution in time of the results for the healthy volunteers and patients. [file 13054_2022_4137_MOESM5_ESM.docx]

**Online Resource 5**

Title: Assessing critical oral comprehension with an eye tracking based innovative device in critically ill patients and healthy volunteers: a cohort study

**Authors**

Laetitia Bodet-Contentin, Hélène Messet-Charrière, Valérie Gissot, Aurélie Renault, Grégoire Muller, Aurélie Aubrey, Pierrick Gadrez, Elsa Tavernier, Stephan Ehrmann

**Evolution in time of the results for the healthy volunteers and patients**


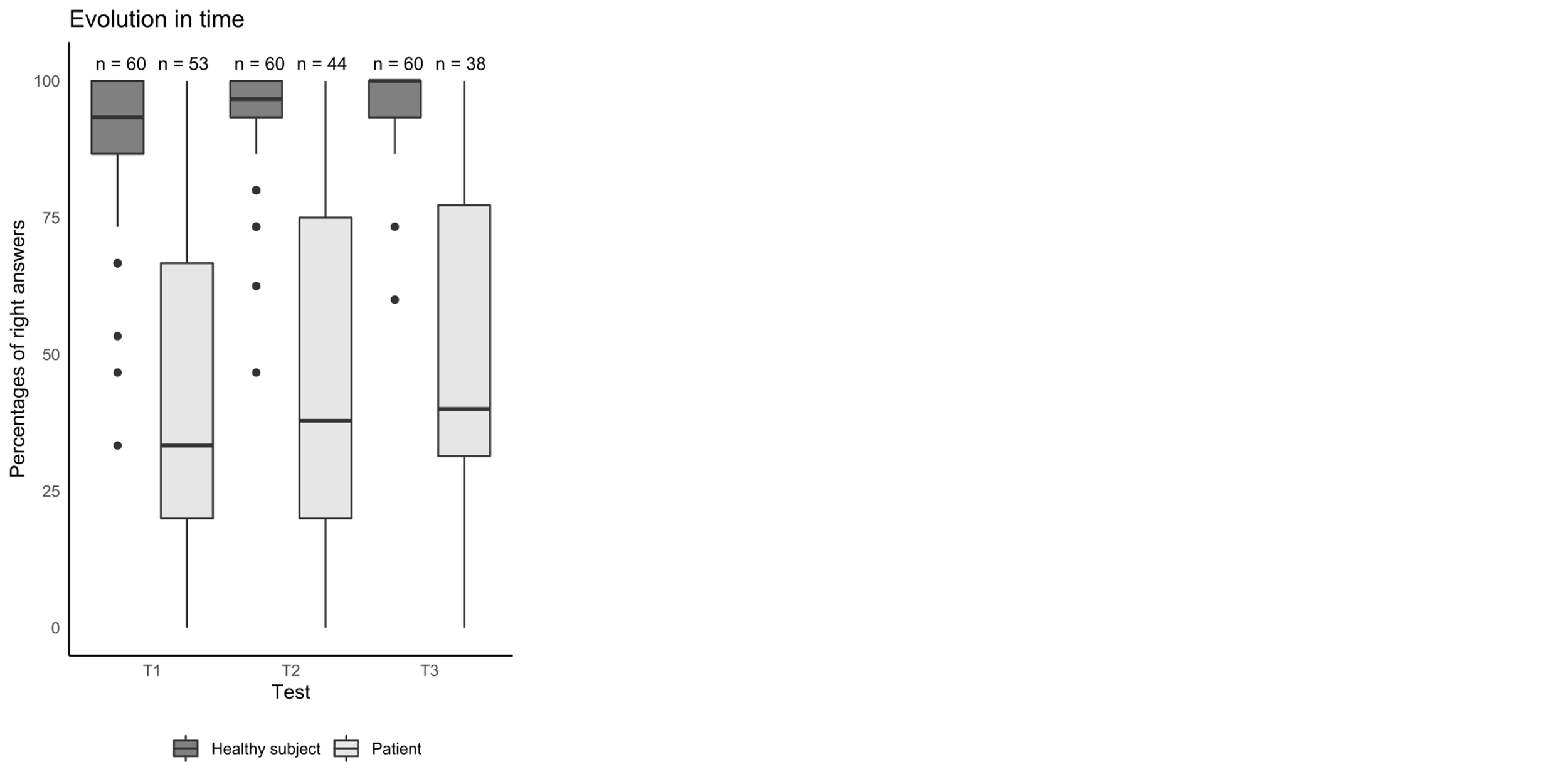


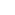

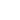

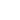


Results according to test 1 (T1), test 2 (T2) and test 3 (T3) realized at three different days for patients, and the same day but in a row for healthy volunteers
