# Supplementary material for: Assessing oral comprehension with an eye tracking based innovative device in critically ill patients and healthy volunteers: a cohort study
Source: Crit Care. 2022 Sep 23;26:288. doi: 10.1186/s13054-022-04137-3 (PMC9508751; doi:10.1186/s13054-022-04137-3)

**Online Resource 6**

Title: Assessing critical oral comprehension with an eye tracking based innovative device in critically ill patients and healthy volunteers: a cohort study

**Authors**

Laetitia Bodet-Contentin, Hélène Messet-Charrière, Valérie Gissot, Aurélie Renault, Grégoire Muller, Aurélie Aubrey, Pierrick Gadrez, Elsa Tavernier, Stephan Ehrmann


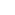

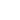

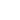


**Results for the test 1 according to the SAPS II score, invasive ventilation, and sedation in patients**


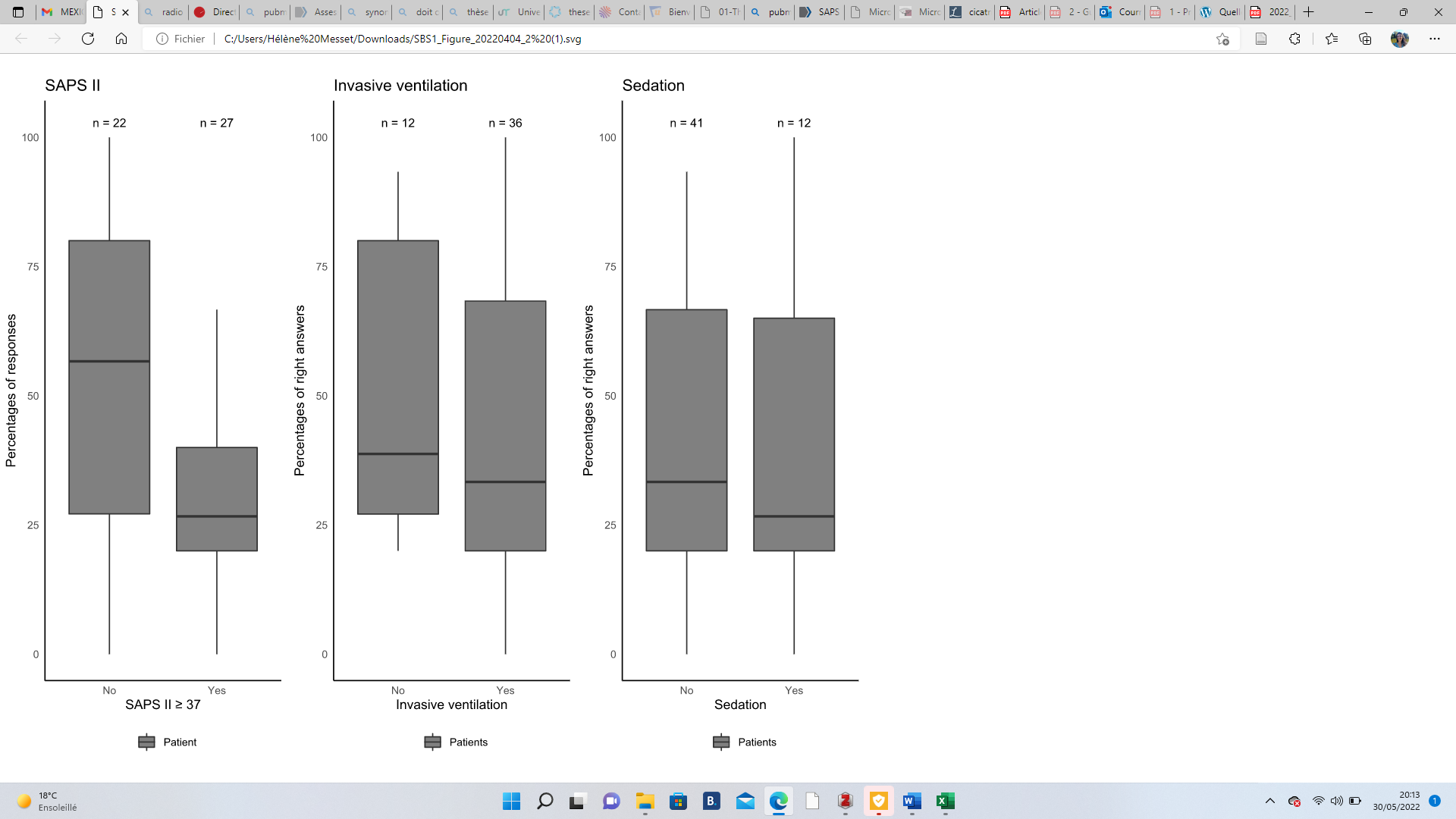


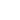

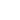

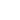

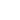

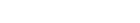

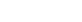

Supplement: Supplementary file 6 — Additional file 6. Results for the test 1 according to the SAPS II score, invasive ventilation, and sedation in patients. [file 13054_2022_4137_MOESM6_ESM.docx]
